# Nonspecific N-terminal tetrapeptide insertions disrupt the translation arrest induced by ribosome-arresting peptide sequences

**DOI:** 10.1016/j.jbc.2024.107360

**Published:** 2024-05-11

**Authors:** Akinao Kobo, Hideki Taguchi, Yuhei Chadani

**Affiliations:** 1 School of Life Science and Technology, Tokyo Institute of Technology, Yokohama, Japan; 2 Cell Biology Center, Institute of Innovative Research, Tokyo Institute of Technology, Yokohama, Japan; 3 Faculty of Environmental, Life, Natural Science and Technology, Okayama University, Okayama, Japan

**Keywords:** SecM, CmlA leader, difficult-to-express proteins, nascent chain, ribosomal stalling, translation arrest, ribosome arrest peptide, translation regulation, SKIK tag

## Abstract

The nascent polypeptide chains passing through the ribosome tunnel not only serve as an intermediate of protein synthesis but also, in some cases, act as dynamic genetic information, controlling translation through interaction with the ribosome. One notable example is *Escherichia coli* SecM, in which translation of the ribosome arresting peptide (RAP) sequence in SecM leads to robust elongation arrest. Translation regulations, including the SecM-induced translation arrest, play regulatory roles such as gene expression control. Recent investigations have indicated that the insertion of a peptide sequence, SKIK (or MSKIK), into the adjacent N-terminus of the RAP sequence of SecM behaves as an "arrest canceler". As the study did not provide a direct assessment of the strength of translation arrest, we conducted detailed biochemical analyses. The results revealed that the effect of SKIK insertion on weakening SecM-induced translation arrest was not specific to the SKIK sequence, that is, other tetrapeptide sequences inserted just before the RAP sequence also attenuated the arrest. Our data suggest that SKIK or other tetrapeptide insertions disrupt the context of the RAP sequence rather than canceling or preventing the translation arrest.

All known life forms on Earth synthesize proteins through the ribosome-mediated polymerization of amino acids. Ribosomes are thought to exhibit high versatility in synthesizing a wide array of proteins with diverse amino acid sequences, meeting the demands of organisms. However, it has been revealed that ribosomes hardly synthesize certain amino acid sequences, such as those containing the basic or acidic amino acid cluster, consecutive proline, or tryptophan repeats (1, 2). This limitation is attributed to the charge and physical constraints of the ribosome tunnel, through which the nascent peptide passes during synthesis, making the synthesis of certain amino acid sequences challenging.

The tunnel structure is essential for ensuring the continuity of translation elongation so that the obstacles mentioned above can be viewed as a kind of fitness cost. Alternatively, numerous cases have been identified where such obstacles are repurposed for physiological functions. For instance, the translation of *Escherichia coli secM* ORF undergoes translation arrest in a manner dependent on its amino acid sequence context (3, 4). The occurrence of such translational arrest senses and determines the expression level of the downstream SecA. Similarly, translation arrest during the synthesis of *E. coli tnaC* or *speFL* also regulates the expression of downstream tryptophanase (TnaA, (5, 6)) or ornithine decarboxylase (SpeF, (7)), respectively. These ribosome arresting peptides (RAP) with regulatory functions have been identified in various bacteria (8, 9, 10, 11, 12) as well as in humans, budding yeast, and plants (13, 14, 15, 16, 17, 18). Although they all depend on the context of the amino acid sequence to arrest translation elongation, each of them exhibits unique amino acid sequences, with little commonality found except in a few instances.

These RAP sequences are likely to be independently acquired during evolution to optimize the expression of target genes or to enhance the function of expressed products. On the other hand, the translation arrest before acquiring physiological function would be simply a risk for protein expression. To mitigate such risks, organisms acquired various translation factors such as elongation factor EF-P (eIF5A in eukaryotes), to promote the translation of problematic amino acid motifs, as exemplified by consecutive proline sequences (19, 20, 21). Nevertheless, even in the presence of EF-P, numerous translation stalling, premature termination, and other noncanonical translations continue to occur (22, 23). Such translation defect would be disadvantageous to merely synthesizing and expressing proteins. Therefore, resolving or preventing these problems during translation elongation would become a valuable means for the large-scale production of various useful proteins.

Recently, Ojima-Kato *et al.* reported a significant enhancement in translation initiation efficiency by inserting the "SKIK" sequence adjacent to the start codon (24). This aligns with findings that the "bulkiness" of the N-terminal amino acid sequence promotes efficient translation (25, 26, 27) or AU-richness of the mRNA sequences (25, 28, 29, 30). Furthermore, they proposed that the SKIK sequence inserted just upstream of the RAPs of SecM or *cmlA* leader (31, 32, 33, 34) cancels translation arrest (35). If the SKIK tagging of the RAP sequence could prevent various kinds of translation arrest as they have shown, it would be an innovative advancement with broad applications. However, their evaluation of the arrest cancellation effect was limited to the synthesis quantity of full-length proteins, and they did not directly evaluate the translation arrest products. Therefore, it was not certain whether SKIK tagging canceled translation arrest.

We aimed to directly investigate the arrest cancellation activity through the SKIK tagging using the established analytical techniques. Our results demonstrate that the SKIK tagging attenuated the translation arrest, however, identical results were obtained even when randomly chosen tetrapeptides were inserted. These findings indicate that the effect of SKIK tagging on translation arrest was not specific to the SKIK sequence. Taking into account the supplementary findings and previously published data (4), we conclude that the peptide tag insertion, including SKIK, adjacent upstream of the RAP perturbs the "invisible" context essential for inducing translation arrest.

## Results and discussion

**Figure 1:**
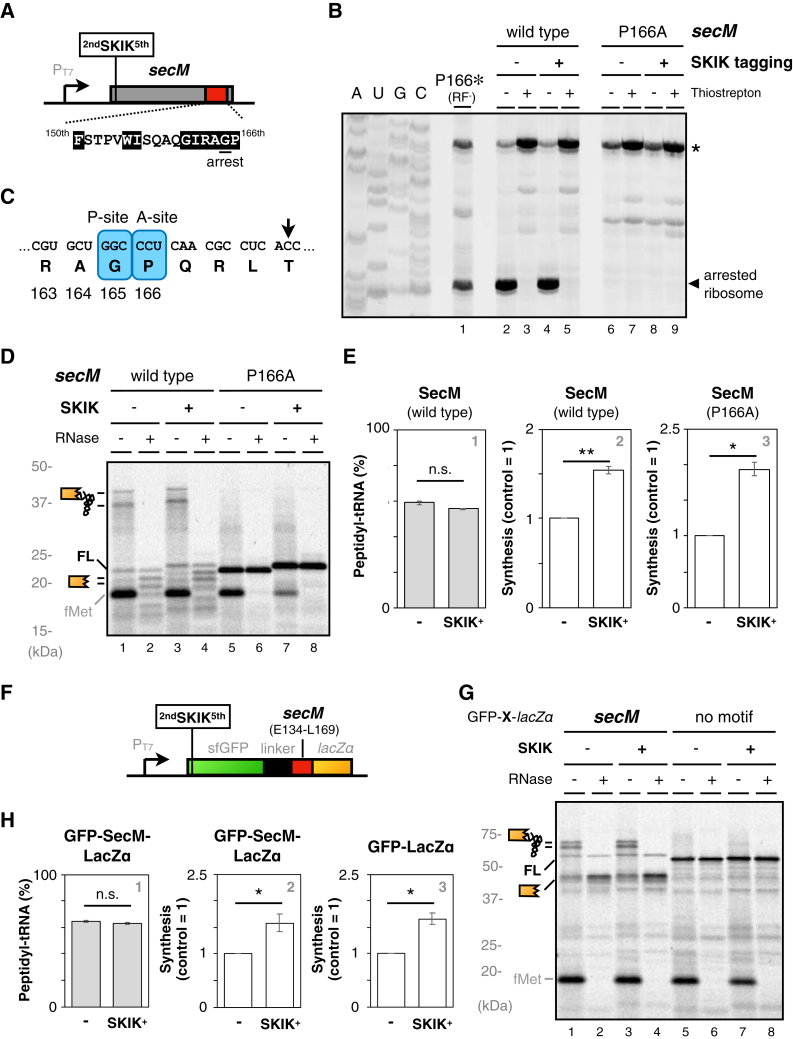
**Re-evaluation of the impact of SKIK tagging on the translation initiation site of *secM* ORF.***A*, schematic of the N-terminal insertion of SKIK tagging. The SKIK sequence was fused with the initiation methionine codon. The ^150^FxxxxWIxxxxGIRAGP^166^ arrest motif of SecM is also indicated. *B*, toeprint analysis. The PURE system mixture was directed with either *secM* (lanes 2 and 3), N-terminally SKIK-tagged *secM* (lanes 4 and 5), *secM*_P166A mutant (lanes 6 and 7) or N-terminally SKIK-tagged *secM*_P166A template (lanes 8 and 9). To discriminate the translation arrest-dependent attenuation of reverse transcription, 200 μg/ml of thiostrepton which inhibits translation elongation was added at the start point where indicated. Reaction mixtures were then subjected to reverse transcription using a downstream, fluorescent primer. Dideoxy sequencing reactions were also primed by the same primer (lanes A, U, G, and C). The *secM* carrying the P166amber mutation was translated in the absence of release factors (RFs) to prepare the ribosomes stalled at the P166 codon for the position marker (lane 1). An asterisk indicates a non-specific signal which is independent of the translation of *secM* mRNA. *C*, schematic illustration of the ribosome stalling site on the *secM* mRNA. The *arrow* indicates the point of reverse transcription interference, and the ribosomal occupancy is shown by the A-site and P-site codons. *D*, the *secM* (lanes 1 and 2), N-terminally SKIK-tagged *secM* (lanes 3 and 4), *secM*_P166A mutant (lanes 5 and 6), or N-terminally SKIK-tagged *secM*_P166A mRNA (lanes 7 and 8) were translated by PURE*frex*, respectively. Translation products were labeled with Cy5-Met-tRNA, as described previously (23). Samples were separated by neutral pH SDS-PAGE with optional RNase A (RN) pretreatment. The peptidyl-tRNA and tRNA-released truncated peptide are schematically indicated. The full-length product (FL) and Cy5-fMet-tRNA (fMet) are also indicated. *E*, Panel 1: The ratio of SecM peptidyl-tRNA, calculated as described in the Experimental procedures. Panels 2 and 3: Relative protein expression from the *secM* or *secM*_P166A mRNA with or without N-terminal SKIK tagging, calculated as described in the Experimental procedures. ∗*p*-value < 0.05, ∗∗*p*-value < 0.01, n.s.: no significant difference (Welch’s *t* test). (#). *F*, schematic of GFP-*secM-lacZα* mRNA. *G*, the GFP-*secM-lacZα* (lanes 1 and 2), N-terminally SKIK-tagged GFP-*secM-lacZα* (lanes 3 and 4), GFP*-lacZα* (lanes 5 and 6), or N-terminally SKIK-tagged GFP*-lacZα* mRNAs (lanes 7 and 8) were translated and analyzed as Figure 1*D*. *H*, panel 1: The ratio of GFP-SecM peptidyl-tRNA, calculated as described in the Experimental procedures. Panels 2 and 3: Relative protein expression from GFP-*secM*-*lacZα* or GFP-*lacZα* mRNA with or without N-terminal SKIK tagging, calculated as described in the Experimental procedures. ∗*p*-value < 0.05, n.s.: no significant difference (Welch’s *t* test). (#). (#) The mean values ± SE estimated from three independent technical replicates are shown.

Ojima-Kato *et al.* concluded the arrest cancelation through SKIK tagging based on the yield of synthesized proteins (35). However, their analysis lacked a direct biochemical evaluation of the arrest indicator, such as a stalled ribosome complex, or nascent peptidyl-tRNA itself. Therefore, to verify the arrest cancelation activity of SKIK tagging, we first examined the effects of SKIK tagging adjacent to the initiation codon and distant from the arrest sequence. As shown in Figure 1*A*, we inserted the SKIK sequence at the N-terminus of SecM. We evaluated the strength of translation arrest by using the reconstituted cell-free translation system, PURE system (PURE*frex* v1.0, (36)). Note that our experiments were conducted using a transcription-translation coupled system, wherein sufficient mRNAs per ribosome were transcribed (Fig. S1).

We first evaluated the occurrence of translation arrest through toeprint analysis, a common method for assessing the amount and position of the arrested ribosomes (15, 16, 37, 38). The insertion of the SKIK tag just after the start codon of *secM* ORF did not reduce the accumulation of reverse transcription products due to the SecM-induced arrest, indicating that the SKIK tagging did not influence the strength of translation arrest at P166 codon (Fig. 1, *B* and *C*). Next, we calculated the strength of translation arrest from the ratio of the accumulated peptidyl-tRNA to the full-length polypeptide (9, 10, 11, 12, 13, 22, 23, 37). The insertion of the SKIK tag did not affect the accumulation of peptidyl-tRNA, consistent with that of the toeprint analysis (Fig. 1, *D* and *E*, panel 1).

The endogenous *secM* sequence contains various motifs that affect the translation arrest, in addition to the RAP sequence (39, 40). To eliminate these factors, we subcloned the RAP sequence, E134 - R169 segment of SecM, which would be sufficient to occupy the ribosome tunnel when SecM peptidyl-tRNA arrests the ribosome, between sfGFP and a *lacZ* fragment (*lacZ*α) (Fig. 1*F*). We found that the insertion of the SKIK tag did not affect translation arrest. These results align with Ojima-Kato's findings that SKIK tags positioned away from the RAP sequence (^150^FxxxxWIxxxxGIRAGP^166^) do not attenuate the translation arrest (Fig. 1, *G* and *H*, panel 1) (35). The constructs with the SKIK tagging increased protein synthesis by around 1.5-fold compared to that without the tagging (Fig. 1*E*, pane 2 and 3, 1*H*, panels 2 and 3).

**Figure 2:**
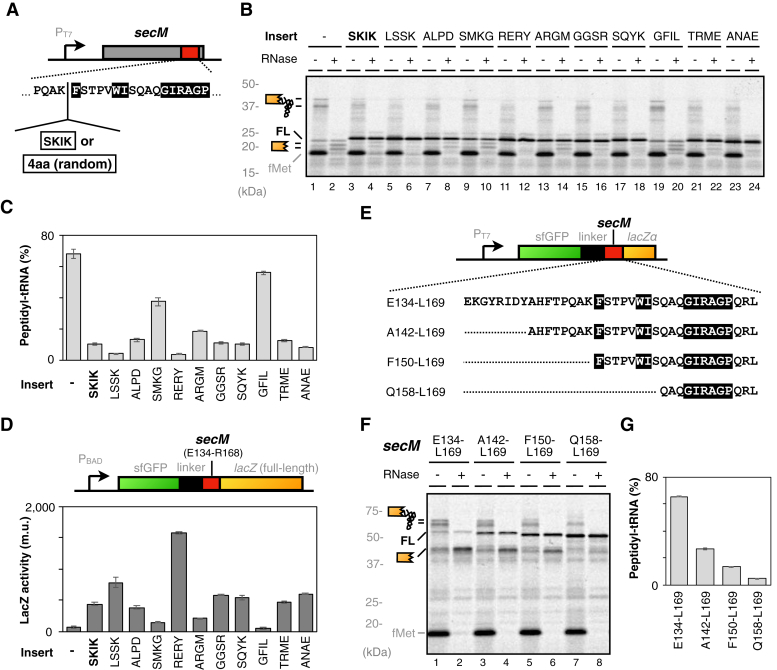
**The effect of SKIK or other tetrapeptide tagging adjacent to the FxxxxWIxxxxGIRAGP ribosome arresting motif of SecM.***A*, schematic of SKIK or tetrapeptide tagging of FxxxxWIxxxxGIRAGP motif. The *white* text on a *black* background indicates the residues whose Ala-substitution disrupts SecM-dependent translation arrest. *B*, the wild-type *secM* mRNA (lanes 1–2), *secM* mRNA with SKIK (lanes 3–4), or other tetrapeptide tagging (lanes 5–24) adjacent to the RAP sequence were translated by PURE*frex* and analyzed as Figure 1*D*. *C*, the ratio of SecM peptidyl-tRNA, calculated from the gel images as represented in Figure 2*B*. (#) *D*, the amount of β-galactosidase expressed from GFP-*secM-lacZ* (full-length) mRNA was evaluated as Miller unit (m.u.) as described previously (23). (#) *E*, schematic of GFP-*secM-lacZα* mRNA and its variants carrying various lengths of *secM* segment. The amino acid sequence of each variant is shown. *F*, the GFP-*secM-lacZα* (lanes 1–2), or its variants carrying various lengths of *secM* segment as indicated (lanes 3–8) were translated and analyzed as Figure 1*D*. *G*, the ratio of GFP-SecM peptidyl-tRNA, calculated from the gel images represented in Figure 2*F*. (#). (#) The mean values ± SE estimated from three independent technical or biological replicates are shown.

Next, we examined the potential arrest-cancelation by SKIK tagging adjacent to the RAP sequence (F150-P166) of SecM. We inserted the SKIK tag just before the F150 residue of *secM* ORF as in the previous study (35) and analyzed the resulting translation product (Fig. 2*A*). The insertion of the SKIK tag reduced the accumulation of the RAP-derived peptidyl-tRNA and increased the synthesis of the full-length product (Fig. 2*B*), consistent with the previous report. To test the specificity of the SKIK sequence in reducing the SecM-induced translation arrest, we replaced the SKIK tag with randomly generated tetrapeptide tags. The results revealed that eight out of the ten inserted sequences weakened translation arrest to an equal or greater extent than the SKIK tag (Fig. 2*C*). This trend was reproduced by a reporter assay in *E. coli* cells (Fig. 2*D*). These results suggest that the tunnel-embedded SKIK sequence does not have a specific effect in canceling translation arrest in its downstream sequences. Instead, there is a possibility that translation arrest is attenuated by other reasons.

The ribosome tunnel accommodates about 30 to 40 amino acids region of the nascent peptide, interacting with the tunnel wall at various sites (1, 2, 41, 42). In addition, arrest sequences other than the RAP of SecM have highlighted the importance of amino acid residues located further away from the peptidyl transferase center than the F150 residue of SecM (9, 10, 11, 18, 43). Therefore, we re-evaluated whether the SecM sequence other than F150 - P166 is involved in the complete occurrence of SecM-dependent arrest. From the comparison between the GFP-SecM-LacZα and its variants carrying longer N-terminal SecM sequence, it appears that the E134-L169 segment contains most of the elements required for full induction of the SecM-dependent translation arrest (Fig. S2). We then deleted the *secM* segment in the GFP-*secM-lacZα* construct as depicted in Figure 2*E* and evaluated their translation arrest activity based on the accumulation of peptidyl-tRNA. As the *secM* sequence was progressively truncated, the accumulation of peptidyl-tRNA was significantly attenuated (Fig. 2, *F* and *G*). Most notably, the ^150^FxxxxWIxxxxGIRAGP^166^ segment, usually regarded as a minimal RAP sequence, was not sufficient to fully induce the SecM-dependent translation arrest. This suggests that, in addition to the amino acid residues identified by alanine scanning analysis, the nascent peptide segment, which precedes the F150 residue and is accommodated within the tunnel, also contributes to the translation arrest. Consistent with this result, Nakatogawa and Ito have already shown that the SecM D140-P166 segment induces stronger translation arrest than the F150-P166 segment (2).

**Figure 3:**
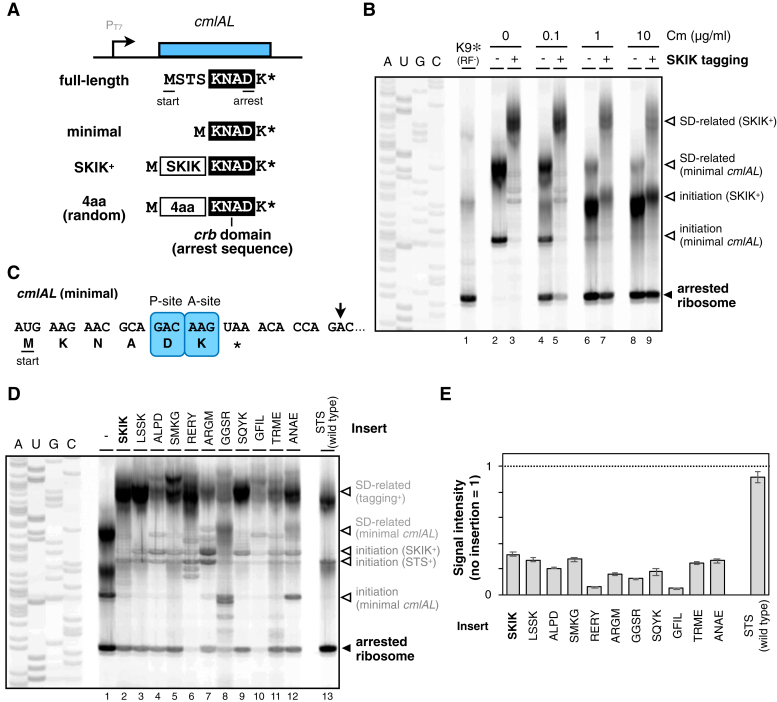
**Evaluation of the impact of tetrapeptide tagging adjacent to *cmlA* leader.***A*, schematics representation of *cmlA* leader (*cmlAL*) and its derivatives used in this study. The SKIK or tetrapeptide tag was inserted between the initiation codon and the "KNAD" ribosome arresting motif of *cmlAL*. *B*, toeprint analysis. The PURE*frex* mixture was directed by the minimal *cmlAL* (even lanes), or adjacently SKIK-tagged *cmlAL* mRNA (lanes 3, 5, 7, and 9), in the presence of the indicated concentration of chloramphenicol (Cm), respectively. Reaction mixtures were then subjected to reverse transcription using a downstream, fluorescent primer. Dideoxy sequencing reactions were also primed by the same primer (lanes A, U, G, and C). The minimal *cmlAL* carrying K9amber mutation was translated in the absence of release factors (RFs) to prepare the ribosomes stalled at the Lys9 codon for the position marker (lane 1). The *black arrow* indicates the ribosome stalled at the ninth Lys codon. The *white* arrows indicate ribosomes stalled at the start codon of the *cmlAL* or its SKIK-tagged variant, respectively, or the signals related to the SD sequence. A representative of three independent experiments is shown. *C*, schematic illustration of the ribosome stalling site on the *cmlAL* mRNA. The *arrow* indicates the point of reverse transcription interference, and the ribosomal occupancy is shown by the A-site and P-site codons. *D*, toeprint analysis of the minimal *cmlAL*, or adjacently SKIK/tetrapeptide-tagged *cmlAL* mRNA as indicated; the minimal *cmlAL* mRNA (lane 1), *cmlAL* mRNA with SKIK (lanes 2), other tetrapeptide tagging (lanes 3–12) adjacent to the RAP sequence, or full-length *cmlAL* (with STS sequence, lane 13). The *black arrow* indicates the ribosome stalled at the ninth Lys codon. The *white arrows* indicate ribosomes stalled at the start codon of the *cmlAL* or its variant, respectively, or the signals related to the SD sequence. *E*, the amount of arrested ribosomes was evaluated from the signal intensity of the gel images as represented in Figure 3*D*. The values normalized by that of the minimal *cmlAL* (no insertion, lane 1 in Fig. 3*D*) were represented. (#). (#) The mean values ± SE estimated from three independent technical replicates are shown.

The arrest cancelation by SKIK tagging was also reported for chloramphenicol (Cm)-dependent translation arrest induced by the *cmlA* leader (*cmlAL*). Following the results with SecM, we investigated whether SKIK sequence-specific arrest cancelation occurs for the *cmlAL* as well. We also employed the minimal *cmlAL*, which exclusively encompasses the conserved *crb* domain (KNAD) among Cm-resistant gene leader sequences (Fig. 3*A*, ref. (31, 32, 33, 34)). Previous studies indicated that the ribosome translating the *cmlAL* ORF is arrested in the presence of sub-inhibitory concentrations of Cm, at Asp8 and Lys9 codons located in the P-site and A-site, respectively (34). Through toeprint analysis, we confirmed that the minimal *cmlAL* arrested the ribosome in the presence of Cm (Fig. 3*B* lanes 4–9, 3*C*), as reported previously (34). Note that the SKIK insertion-dependent arrest cancelation was observed in the presence of 0.1 μg/ml concentration of Cm, but not when Cm concentration was excessive (Fig. 3*B*, lanes 4–9). Therefore, subsequent experiments were conducted in the presence of 0.1 μg/ml concentration of Cm.

Next, the same set of 10 tetrapeptide tags used in the SecM analysis was introduced into the minimal *cmlAL*, and their effects on arrest cancelation were assessed. The toeprint experiment in Figure 3*D* demonstrated that, akin to SecM, the insertion of tetrapeptide tags, including SKIK, abolished translation arrest in the *cmlAL* (Fig. 3, *D* and *E*). This finding implies that the arrest cancelation achieved by SKIK-tagging of the *cmlAL* is not specific to the SKIK sequence, as in the case of *secM*. The lack of significant effect of GFIL on SecM translation arrest, compared to its cancellation of CmlAL, may suggest a lower tolerance of CmlAL to insertions due to its minimal amino acid motif for the arrest occurrence. Conversely, when the native "STS" sequence of the *cmlAL* was reintroduced, translation arrest was not affected (Fig. 3*D* lane 13, 3*E*). This indicates that the native STS sequence of the *cmlAL* contributes to translation arrest in a manner distinct from the conventional "arrest motif" - it does not impede translation arrest.

## Conclusion

Based on the findings from N-terminal tagging of the RAP (arrest) sequences of SecM and CmlAL, we draw the following conclusions.

1. The arrest cancellation by SKIK tag inserted "N-terminally adjacent to" the RAP sequences, is not exclusive to SKIK. This suggests that the observed effect is not contingent on a specific amino acid sequence but arises from disrupting the "context" necessary for SecM- or CmlAL-induced translation arrest. Consequently, the peptide tag insertion leads to an attenuation of translation arrest and an enhancement of full-length product synthesis.
2. Robust and complete induction of SecM-induced translation arrest requires more than the ^150^FxxxxWIxxxxGIRAGP^166^ motif identified through alanine scanning. The nascent peptide segment of SecM, which is accommodated within the exit tunnel and precedes the F150 residue, also has an impact (consistent with the previous study by Nakatogawa and Ito (4)).

Recently, Gersteuer and colleagues presented the structure of the ribosome arrested by the "full-length" SecM nascent peptide (44). In this structure, residues 132 to 149 of SecM also interact with the exit tunnel for robust stalling, consistent with our biochemical results (Figs. 2, *E*–*G* and S2). In addition, Judd and colleagues reported that the non-conserved residues of TnaC could also affect its RAP function (45). Based on these findings and our studies, we raise a caveat about the term "arrest sequence/motif", commonly referred to as the amino acid sequence responsible for inducing translation arrest. The amino acid residues identified through alanine scanning and sequence analyses undoubtedly represent essential elements for translation arrest. However, as demonstrated in this paper and previous studies, translation arrest is not solely induced by the "arrest sequence/motif" but could be modulated by the other region(s) of the nascent peptide (39, 40, 43, 44, 45). We underscore that the "arrest sequence/motif" is necessary but, in some cases, not sufficient for the induction of translation arrest.

## Experimental procedures

### *E. coli* strains, plasmids, and primers

*E. coli* strain BW25113 {Δ(*araD-araB*)567, Δ*lacZ4787*(::*rrnB*-3), λ-, *rph*-1, Δ(*rhaD*-*rhaB*)568, *hsdR514*} was used as the experimental standard strain. Plasmids and oligonucleotides used in this study are listed in Tables S1 and S2, respectively. Plasmids were constructed using standard cloning procedures and detailed schemes are summarized in Table S1. The sequence files of the constructed plasmids are available in the Mendeley repository (https://doi.org/10.17632/5db6jbtrcy.1).

### *In vitro* translation and product analysis

The coupled transcription-translation reaction was performed using PURE*frex* 1.0 (GeneFrontier) at 37 °C for 30 min. N-terminal fluorescent labeling of PURE*frex* translation products was performed using pre-charged Cy5-Met-tRNA, as described previously (23).

DNA templates were prepared by PCR, as summarized in Table S3. The reaction was stopped by dilution into an excessive volume of 5% TCA. After standing on ice for at least 10 min, the samples were centrifuged for 3 min at 4 °C, and the supernatant was discarded by aspiration. The precipitates were then vortexed with 0.9 ml of acetone, centrifuged again, and dissolved in SDS sample buffer (62.5 mM Tris-HCl, pH 6.8, 2% SDS, 10% glycerol, 50 mM DTT) that had been treated with RNAsecure (Ambion). Finally, the sample was divided into two portions, one of which was incubated with 50 μg/ml of RNase A (Promega) at 37 °C for 30 min, and separated by a WIDE Range SDS-PAGE system (Nakalai Tesque). Images were visualized and analyzed by FLA7000 image analyzer (GE Healthcare) using a 635 nm excitation laser and LPR emission filter.

### Quantitative real-time PCR

After the addition of the DNA template into the PURE*frex* mixture (5 μl) and subsequent incubation at 37 °C for 30 min for the coupled transcription-translation, 0.25 unit of DNase I (TaKaRa) was added and further incubated at 37 °C for 30 min. The total RNA in the mixture was extracted using the RNeasy MinElute Cleanup kit (QIAGEN) according to the manufacturer's instructions. Quantitative real-time PCR was performed using the Luna Universal One-Step RT-qPCR Kit (New England Biolabs) and the M x 3000P Real-Time QPCR System (Agilent Technologies). The RT-qPCR reaction to determine the amount of *secM* mRNA was conducted using oligonucleotides AK0331 and AK0332 (Table S2) with the following settings: 10 min at 55 °C and 1 min at 95 °C followed by 40 cycles of 10 s at 95 °C and 35 s at 60 °C. The *secM* mRNA for calibration was *in vitro* transcribed from the DNA template by using the CUGA *in vitro* transcription kit (Nippon Gene), followed by purification with the RNeasy MinElute Cleanup kit. The concentration of the purified mRNA was determined using the Qubit 4 Fluorometer (Invitrogen).

### Toeprint analysis

Toeprint analysis was performed as described previously (22, 38). *In vitro* translation reaction sample was mixed with an equal volume of reverse transcription mixture [50 mM HEPES-KOH pH 7.6, 100 mM potassium glutamate, 2 mM spermidine, 13 mM magnesium acetate, 1 mM DTT, 2 μM fluorescently labeled oligonucleotide (pe-lacZ-N-rv with Alexa 647 at 5′-terminus), 40 μM of each of dNTPs, 10 unit/μl ReverTra Ace (Toyobo)] and incubated at 37 °C for 10 min. The reverse transcription products were purified by NucleoSpin Gel and PCR clean-up kit equilibrated with NTC buffer (Macherey-Nagel). Dideoxy DNA sequencing samples were prepared using Thermo Sequenase DNA polymerase (Cytiva) and the same templates and primer (pe-lacZ-N-rv) as used for toeprint analysis. Samples were subjected to 8% polyacrylamide-7 M urea-TBE gel electrophoresis. Fluorescent images were visualized and analyzed by Amersham Typhoon scanner RGB system (GE healthcare) using a 635 nm excitation laser and LPR emission filter. The final 100 μg/ml of thiostrepton was added beforehand if indicated.

### β-galactosidase assay

*E. coli* cells harboring the *lacZ* reporter plasmid were grown overnight at 37 °C in LB medium supplemented with 100 μg/ml ampicillin. On the next day, they were inoculated into fresh LB medium containing 2 × 10^−3^% arabinose and 100 μg/ml ampicillin and were grown at 37 °C for ∼2.5 h (A_660_ = ∼0.6). Afterward, 20 μl portions were subjected to a β-galactosidase assay as described (23). The average of three independent experiments is presented with a standard error (SE) value.

### Quantification of the signals from gel images

The ratio of the translation-completed chain (full-length: FL) against the polypeptidyl-tRNAs (pep-tRNAs), which signifies the occurrence of translational arrest in the total *in vitro* translation products, was calculated from Cy5-fluorescence proportion of “FL” and “pep-tRNA” among the samples without RNase-treatment. Signal intensity was quantified by the Multi Gauge software (Fujifilm), and the amount of peptidyl-tRNA (in %) was obtained by the following formula.

$$\text{peptidyl}-\text{tRNA}\;\left(in\;vitro\right)=100^{*}\left(\text{pep}-\text{tRNA}\right)\;/\;\left[\left(\text{FL}\right)+\left(\text{pep}-\text{tRNA}\right)\right]$$

The protein synthesis ratio was obtained by the following formula.

$$\text{synthesis}\;\text{ratio}=\left[\left\{\text{pep}-\text{tRNA}\left({\text{SKIK}}^{+}\right)\right\}+\left\{\text{FL}\left({\text{SKIK}}^{+}\right)\right\}\right]\;/\;\left[\left\{\text{pep}-\text{tRNA}\left(\text{no}\;\text{SKIK}\right)\right\}+\left\{\text{FL}\left(\text{no}\;\text{SKIK}\right)\right\}\right]$$

The average of three independent experiments is presented with a standard error (SE) value.

### Generation of random tetrapeptide sequences

Random DNA sequences encoding tetrapeptide tags were generated by using Random DNA Sequence Generator (http://www.faculty.ucr.edu/∼mmaduro/random.htm) with the following settings: Size of DNA in bp: 12, GC content: 50. 50. Generated sequences including one or more in-frame stop codons were omitted.

## Data availability

Raw data files are available in the Mendeley Data repository (https://doi.org/10.17632/5db6jbtrcy.1).

## Supporting information

This article contains supporting information (46, 47).

## Conflict of interest

The authors declare that they have no known competing financial interests or personal relationships that could have appeared to influence the work reported in this paper.
