# Supplementary material for: Nonspecific N-terminal tetrapeptide insertions disrupt the translation arrest induced by ribosome-arresting peptide sequences
Source: J Biol Chem. 2024 May 11;300(6):107360. doi: 10.1016/j.jbc.2024.107360 (PMC11190716; doi:10.1016/j.jbc.2024.107360)
Supplement: Supporting Data [file mmc1.pdf]

Supporting Information for

Non-specific N-terminal tetrapeptide insertions disrupt the translation arrest induced by ribosome arresting peptide sequences.

Akinano Kobo[1], Hideki Taguchi[1,2,*] and Yuhei Chadani[3,*]

[1] School of Life Science and Technology, Tokyo Institute of Technology, Yokohama 226-8503, Japan
[2] Cell Biology Center, Institute of Innovative Research, Tokyo Institute of Technology, Yokohama 226-8501, Japan
[3] Faculty of Environmental, Life, Natural Science and Technology, Okayama University, Okayama 700-8530, Japan.

*Correspondence to Yuhei Chadani (ychadani@okayama-u.ac.jp) and Hideki Taguchi (taguchi@bio.titech.ac.jp)

Supplementary Figures S1 and S2

Figure S1. Quantification of mRNA within the *in vitro* transcription-translation coupled system.

Figure S2. Contribution of the N-terminal segment of SecM to the ribosome arresting function.

Supplementary Tables S1-S4 (in a separate Excel file)

Table S1. Plasmids used.

Table S2. Oligonucleotides used.

Table S3. PCR scheme to prepare the template DNA for in vitro translation.

Table S4. Raw data plotted in the figures.

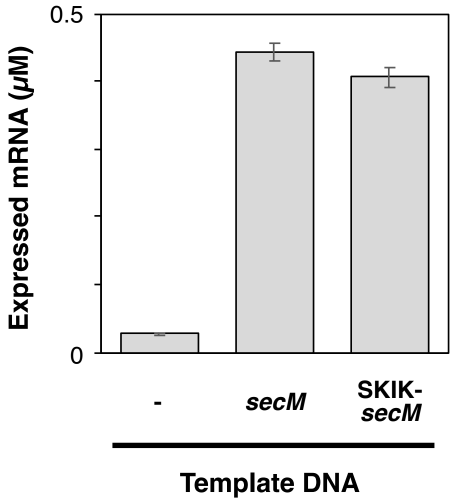

Supplementary Figure 1. Quantification of mRNA within the *in vitro* transcription-translation coupled system.

The quantitative real-time PCR was conducted on the RNA fraction in the PURE*frex* reaction mixture, as described in the Experimental Procedures. The mean values ±SE values of the quantified concentration of *secM*, N-terminally SKIK-tagged *secM* mRNA, or control experiment without any DNA template were plotted, respectively.

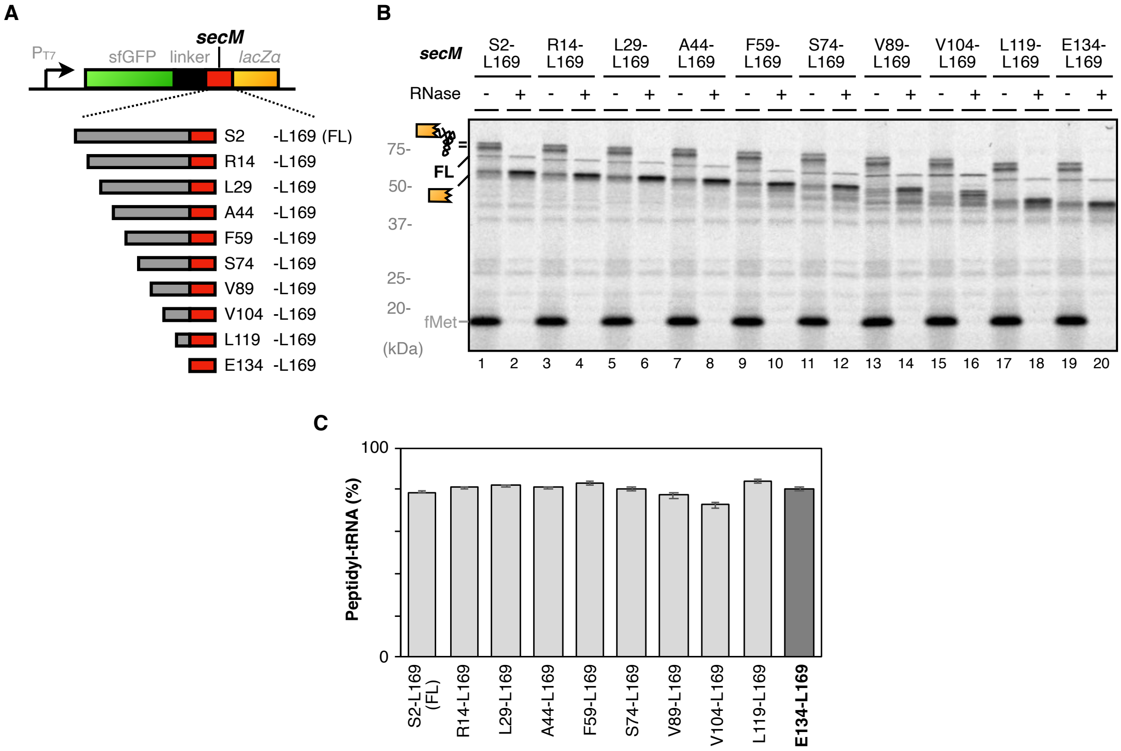

Supplementary Figure 2. Contribution of the N-terminal segment of SecM to the ribosome arresting function.

A. Schematic of GFP-*secM-lacZ*α mRNA and its variants carrying various lengths of *secM* segment.

B. A series of GFP-*secM-lacZ*α variants carrying various lengths of the *secM* segment were translated and analyzed as **Fig. 1D**. The peptidyl-tRNA and tRNA-released truncated peptide are schematically indicated. The full-length product (FL) and Cy5-fMet-tRNA (fMet) are also indicated.

C. The ratio of GFP-SecM peptidyl-tRNA, calculated from the gel images represented in **Fig. S2B**. The mean values ±SE estimated from three independent technical replicates are shown.
